# No evidence for increased prevalence of colorectal carcinoma in 399 Dutch patients with Birt-Hogg-Dubé syndrome

**DOI:** 10.1038/s41416-019-0693-1

**Published:** 2019-12-20

**Authors:** Irma van de Beek, Iris E. Glykofridis, Rob M. F. Wolthuis, Hans J. J. P. Gille, Paul C. Johannesma, Hanne E. J. Meijers-Heijboer, R. Jeroen A. van Moorselaar, Arjan C. Houweling

**Affiliations:** 1 Amsterdam UMC, Vrije Universiteit Amsterdam, Clinical Genetics, De Boelelaan, 1117 Amsterdam, Netherlands; 2 Amsterdam UMC, Vrije Universiteit Amsterdam, Clinical Genetics and Cancer Center Amsterdam, De Boelelaan, 1117 Amsterdam, Netherlands; 3 Amsterdam UMC, Vrije Universiteit Amsterdam, Clinical Genetics and Cancer Center Amsterdam, De Boelelaan, 1117 Amsterdam, Netherlands; 4 Amsterdam UMC, Vrije Universiteit Amsterdam, Clinical Genetics, De Boelelaan, 1117 Amsterdam, Netherlands; 5 Amsterdam UMC, Vrije Universiteit Amsterdam, Department of Pulmonary Diseases, De Boelelaan, 1117 Amsterdam, Netherlands; 6 Amsterdam UMC, Vrije Universiteit Amsterdam, Clinical Genetics and Cancer Center Amsterdam, De Boelelaan, 1117 Amsterdam, Netherlands; 7 Amsterdam UMC, Vrije Universiteit Amsterdam, Urology, De Boelelaan, 1117 Amsterdam, Netherlands; 8 Amsterdam UMC, Vrije Universiteit Amsterdam, Clinical Genetics, De Boelelaan, 1117 Amsterdam, Netherlands

**Keywords:** Genetics research, Colorectal cancer, Cancer genetics

## Abstract

**Background:**

Previously, it has been suggested that colorectal polyps and carcinomas might be associated with Birt-Hogg-Dubé syndrome. We aimed to compare the occurrence of colorectal neoplasms between Dutch patients with Birt-Hogg-Dubé syndrome and their relatives without Birt-Hogg-Dubé syndrome.

**Methods:**

In all, 399 patients with a pathogenic *FLCN* mutation and 382 relatives without the familial *FLCN* mutation were included. Anonymous data on colon and rectum pathology was provided by PALGA: the Dutch Pathology Registry.

**Results:**

No significant difference in the percentage of individuals with a history of colorectal carcinoma was found between the two groups (3.6% vs 2.6%, *p* = 0.54). There was also no significant difference between the age at diagnosis, diameter, differentiation and location of the colorectal carcinomas. Significantly more individuals with Birt-Hogg-Dubé syndrome underwent removal of colorectal polyps (12.2% vs 6.3%, *p* = 0.005). However, there was no significant difference between the number of polyps per person, the histology, grade of dysplasia and location of the polyps.

**Conclusion:**

Our data do not provide evidence for an increased risk for colorectal carcinoma in Birt-Hogg-Dubé syndrome, arguing against the need for colorectal surveillance. The difference in polyps might be due to a bias caused by a higher number of colonoscopies in patients with Birt-Hogg-Dubé syndrome.

## Background

Birt-Hogg-Dubé syndrome (BHD) is a genodermatosis characterised by benign skin lesions called fibrofolliculomas, lung cysts, pneumothorax and an increased risk for renal tumours.^1–3^ BHD is caused by mutations in the *FLCN* gene, of which the majority are truncating loss of function mutations.^4^ In the first report of BHD, two siblings with perifollicular fibromatosis were described. One of them had several colon polyps and an incipient carcinoma (currently considered a polyp with high-grade dysplasia) and an association between the skin phenotype and the colon neoplasms was suggested by the authors.^5^ Since then, colon polyps and colorectal carcinoma (CRC) have been reported in multiple patients with BHD.^6–10^ However, only limited data are available from larger cohorts and no study has confirmed the association between BHD and colon neoplasms with significant statistical power to date. Only one study evaluated the association in a structured manner by comparing a group of 111 BHD patients with 112 of their family members without BHD and found that CRCs had occurred in three and zero of the two groups respectively, a non-significant difference. In addition to the retrospective analysis of patient files, colonoscopies were performed prospectively in a selected number of patients and their family members and in both groups 18% of the individuals had colon polyps.^1^ Nahorski and colleagues evaluated 149 patients with BHD, of which five had a history of CRC. Interestingly, all five patients carried the same germline mutation with a duplication in the poly (C)_8_ tract of the *FLCN* gene, suggesting a possible genotype-phenotype relation.^11^ To further explore the relation between germline *FLCN* mutations and CRC, the *FLCN* gene was analysed in 50 patients with familial CRC without an identifiable genetic cause. No germline *FLCN* mutations were detected.^11^ Another approach to assess a potential correlation between *FLCN* and CRC has been the analysis of the *FLCN* gene in sporadic CRCs. The majority of studies using this approach focused on frameshift mutations in the hypermutable poly (C)_8_ tract in exon 11 of the *FLCN* gene. Germline mutations in the poly (C)_8_ tract are a frequent cause of BHD.^12,13^ A minority of CRCs show microsatellite instability (MSI), meaning that repetitive sequences in the genome are prone to mutations.^14–16^ No mutations in the poly (C)_8_ tract were detected in microsatellite stable (MSS) CRCs (*n* = 110) in two reports.^11,17^ In microsatellite instable CRCs, somatic poly (C)_8_ tract mutations were detected in 5 out of 32, 7 out of 30 and 0 out of 7 CRCs.^11,17,18^ No loss of the second *FLCN* allele was observed in these tumours, whereas loss of heterozygosity has been observed in the majority of *FLCN*-related renal cell carcinomas.^19^ A role for these mutations in the development of MSI CRC cannot be completely ruled out, but they are more likely to be passenger mutations caused by the MSI. Somatic mutations in the poly (C)_8_ tract of *FLCN* have also been observed in endometrial and gastric carcinoma with MSI, supporting the notion that these are passenger mutations, since these tumours have not been reported to be associated with BHD.^20,21^ In a smaller number of sporadic CRCs, the whole *FLCN* gene has been sequenced and only a few somatic and/or germline missense variants of unknown significance were detected providing further evidence for the limited impact of *FLCN* in CRC, if present al all.^17,18,22^ Whole exome sequencing of MSS CRCs in African Americans has detected truncating somatic *FLCN* mutations in 3 out of 103 tumours, whereas no mutations were detected in 129 MSS CRCs from Caucasian patients. The authors propose that *FLCN* might be a driver gene for CRC in the African American population, but further studies are necessary to confirm this.^23^ As far as we know, only one colorectal tumour from a BHD patient has been assessed for a second hit in *FLCN*. By sequencing tumour DNA and normal tissue, loss of heterozygosity at the locus of the germline *FLCN* mutation was shown.^24^ Since only one tumour was tested, this observation does not prove a causal role of loss of *FLCN* in the development of this tumour. It might also be a passenger event or an effect of the role of another tumour suppressor gene on chromosome 17p, such as *TP53*.

It has been suggested that periodic colonoscopy might be considered in a subgroup of families with BHD.^25^ Currently, the Dutch guidelines on BHD recommends to consider colorectal surveillance every 5 years starting at age 45 for BHD patients in families that have at least one patient with both BHD and CRC in the family, irrespective of the age at diagnosis of CRC.^26^ However, based on the available literature, it is difficult to determine if colorectal surveillance is beneficial to BHD patients, and whether it can be of benefit for all patients or just a specific subgroup of patients. In this study, we aimed to study the association of BHD with colorectal neoplasms by comparing the occurrence of colon polyps and CRC between Dutch BHD patients and their family members without BHD.

## Methods

### Patients

Individuals with and without BHD were selected based on data from molecular testing since most, but not all DNA testing of Dutch patients with BHD has been performed in the diagnostic laboratory of the Amsterdam UMC, location VUmc. Until July 2016, 113 families with a pathogenic *FLCN* mutation were identified. Index patients and their family members who underwent DNA-testing were selected for inclusion. Four individuals (with and without BHD) had also been tested positive for Lynch syndrome and were excluded. We were able to include 399 BHD patients (*FLCN*^MUT^ group) and 382 of their family members who were tested negative for the familial *FLCN* mutation (*FLCN*^WT^ group). Data were collected through PALGA, the nationwide network and registry of histo- and cytopathology in the Netherlands, with nationwide coverage of all academic and non-academic centres since 1991, after approval by their scientific committee.^27^ We provided PALGA with pseudonymised patient data based on name, date of birth and gender, via a Trusted Third Party. These were linked to the data in the PALGA database. Data on pathology reports of colon and rectum, the age of the patients at time of diagnosis, a small set of clinical information and the conclusions of the pathology reports were provided by PALGA. All information that could lead to identification of the individuals (including the pseudonym) was removed. Since all data were anonymous for us, no informed consent of the patients was necessary.  The data were subdivided into subgroups: the *FLCN*^*MUT*^ and the *FLCN*^*WT*^ group, and within the *FLCN*^*MUT*^ group specifically patients with and without a mutation in the poly (C)_8_ tract. After the process of pseudonymisation, we could not link the results to other clinical data of the patients anymore, such as their pedigrees or their BHD phenotype.

### Clinical analysis

All pathology reports on CRC and polyps were evaluated. Most reports on CRCs included the location, size and differentiation of the tumour. TNM stage was not mentioned routinely. Most of the reports on polyps included the location and histology and when applicable, the grade of dysplasia. CRC and polyps were considered distal when they were located in the rectum, rectosigmoid, sigmoid or colon descendens and they were considered proximal at all other locations. The differentiation grade of the carcinomas was divided in three groups; well/moderate (well and well/moderate), moderate (moderate) and poor (moderate/poor and poor). The grade of dysplasia of the polyps was divided in three groups; no/mild (no, low grade or low/moderate grade of dysplasia), moderate (moderate grade and moderate/high grade of dysplasia) and severe (high grade of dysplasia and suspicious for CRC but no CRC diagnosed). The indications for colonoscopies that detected CRC and polyps were scored based on the clinical information provided in the reports. Since patients with polyps are usually advised to undergo subsequent colonoscopies, polyps found with colonoscopies within 5 years after finding a polyp or CRC, were considered to have been detected because of the same indication as the first colonoscopy.

### Statistical analysis

To compare variables between the *FLCN*^*MUT*^ and *FLCN*^*WT*^ groups, *t*-tests were used for continuous, normally divided variables, Mann–Whitney U test was used for discrete data (the number of polyps) and Fishers exact test for categorical variables. SPSS software was used for the analysis (IBM Corp. Released 2013. IBM SPSS Statistics for Windows, Version 22.0. Armonk, NY: IBM Corp).

## Results

### General characteristics

No significant differences were present in the basic demographics of the two groups. The percentage of males was 47.1 in the *FLCN*^*MUT*^ group and 42.4 in the *FLCN*^*WT*^ group (*p* = 0.20). The mean age as of 1 July 2016 was 54.5 and 52.2 in the *FLCN*^*MUT*^ and *FLCN*^*WT*^ group respectively (*p* = 0.06). In fact, this should be considered a virtual age, because we did not have data about whether individuals were alive or deceased.

### Colorectal carcinoma

**Fig. 1:**
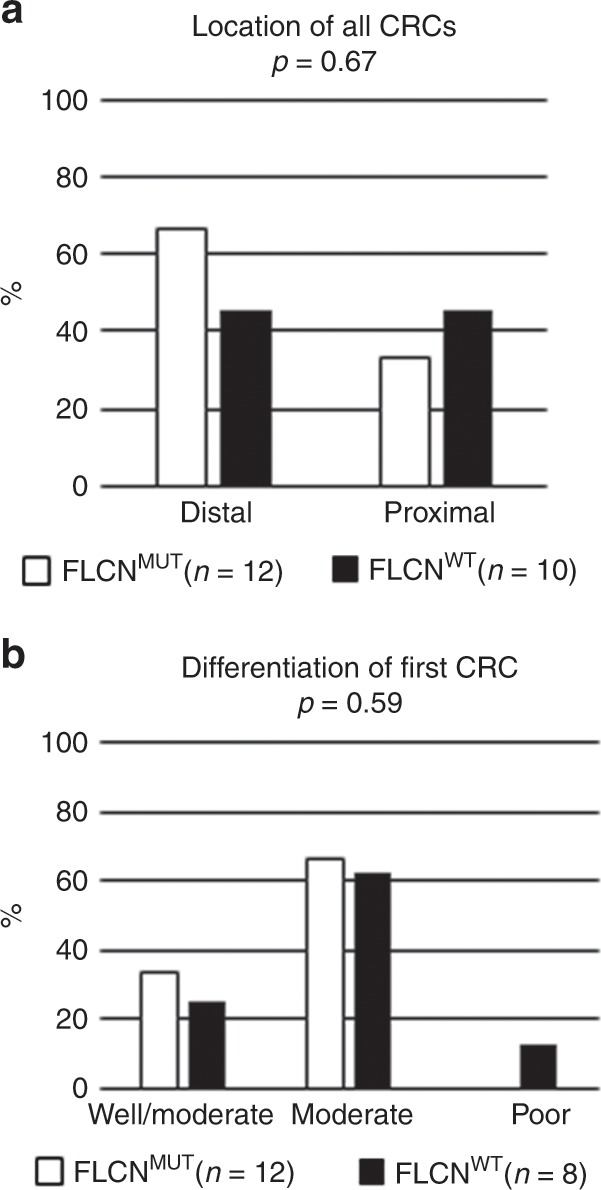
Characteristics of CRCs in the FLCNMUT and FLCNWT group. Location (**a**) and differentiation (**b**) of CRCs in the *FLCN*^*MUT*^ and *FLCN*^*WT*^ group.

Overall, 24 colorectal carcinomas had occurred in 22 individuals. There was no significant difference in the percentage of individuals with CRC in the *FLCN*^*MUT*^ and *FLCN*^*WT*^ group (3.3% vs 2.4%, *p* = 0.52). There was also no significant difference between the groups regarding age at the first CRC, the diameter and differentiation of the first CRC and location of the CRCs (Table 1 and Fig. 1). Dot plots of the age and diameter of the first CRC are shown in Fig. S1, additional data per individual are shown in Tables S1 and S2.

**Table 1:** CRCs and polyps in individuals with and without BHD.

	*FLCN*^*MUT*^	95% CI	*FLCN*^*WT*^	95% CI	*p*-value
Individuals with CRC (*n*)	13 (3.3%)	1.5–5.0%	9 (2.4%)	0.8–3.9%	0.52
Individuals with multiple CRC (*n*)	1 (0.3%)		1 (0.3%)		1
Mean age at first CRC	61.3	54.7–67.9	69.8	61.0–78.5	0.09
Diameter of first CRC (mm)	39.8 (*n* = 11)	27.0–52.6	46.7 (*n* = 6)	33.9–59.4	0.44
Individuals with at least 1 polyp (*n*)	47 (11.8%)	8.6–15.0%	22(5.8%)	3.4–8.1%	**0.004**
Individuals with at least 1 adenoma (*n*)	38 (9.5%)	6.6–12.4%	19 (5.0%)	2.8–7.2%	**0.019**
Individuals with at least 1 villous adenoma (*n*)	1 (0.3%)		1 (0.3%)		1
Median number of polyps per individual	2 (1–29)		2 (1–6)		0.64
Mean age at first polyp	59.9	56.9–62.8	60.3	54.1–66.5	0.89

Statistically significant *p*-values are in bold

### Colorectal polyps

**Fig. 2:**
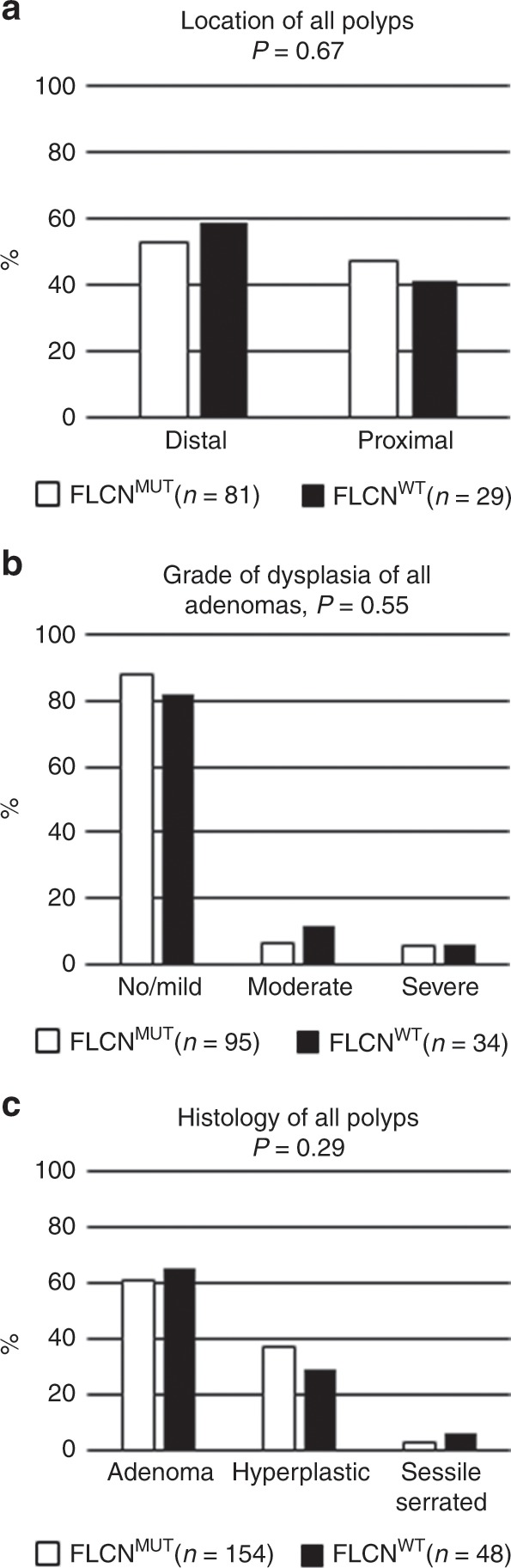
Characteristics of polyps in the FLCNMUT and FLCNWT group. Location (**a**), grade of dysplasia (**b**) and histology of polyps (**c**) in the *FLCN*^*MUT*^ and *FLCN*^*WT*^ group.

The data on polyps are shown in Table 1 and Fig. 2. Significantly more individuals in the *FLCN*^*MUT*^ group were diagnosed with at least 1 polyp (11.8% vs 5.8%, *p* = 0.004). However, the median number of polyps per individual did not differ between the groups, nor did the type of polyps, grade of dysplasia, and location of the polyps. In both groups, only one villous polyp had occurred. Dot plots of the number of polyps per person and age at the first polyp are shown in Fig. S2, additional data per individual are shown in Tables S1 and S2.

### Indications for colonoscopies

Only three reports in the *FLCN*^*MUT*^ group and none in the *FLCN*^*WT*^ group mentioned the indication for the colonoscopy that detected the first CRC. One patient with BHD and CRC underwent the colonoscopy because of skin lesions (age 39), one because of an increased risk for CRC (not specified, age 60) and one because of rectal bleeding (age 67). Considering all colonoscopies, six patients with BHD were examined because of their BHD (as presumably advised by their geneticist). In 12 patients with BHD, all colonoscopies were performed for other indications, including: a positive CRC population screening test, gastro-intestinal symptoms, familial CRC, abnormalities in the colon on imaging and inflammatory bowel disease.

### Evaluation of patients with mutations in the poly (C)_8_ tract

There were 24 patients with BHD from 15 families with a germline mutation in the poly (C)_8_ tract. Two families had a deletion of one cytosine and 13 families had a duplication of one cytosine. Colon polyps were resected in 2/24 patients (8.3%), they each had 1 and 2 polyps, respectively. CRC had occurred in none of these patients.

## Discussion

We performed the largest clinical study to date addressing the association between BHD and colorectal neoplasms. Colorectal polyps are relatively common neoplasms that, in the majority of cases, do not cause clinical symptoms. Among healthy asymptomatic patients undergoing colonoscopy, adenomas are expected to be detected in ≥ 25% of men and ≥ 15% women over the age of 50 years.^28^ Surveillance with colonoscopy will therefore lead to removal of polyps which would otherwise not have been detected.

In the Netherlands, colonoscopies are recommended every 5 years starting at age 45 for BHD patients from families that have at least one member with both BHD and CRC. These guidelines are based on early reports that indicated an association between BHD and CRC. However, current literature does not support a strong causal link between *FLCN* mutations and CRC, and the benefit of colonoscopies for BHD patients, even for a subset with familial occurrence of CRC, has not been demonstrated. In order to re-evaluate the recommendations for BHD patients, we compared the prevalence of CRC in 399 patients with a pathogenic *FLCN* mutation and 382 relatives without the familial *FLCN* mutation. For this study, only anonymised data were available which limited the cross-correlation of indication records, normal colonoscopies and other clinical information that also may have provided more insight into interfamilial variations.

We chose the anonymised study design because obtaining the required informed consent from all patients and collecting the hospital records would have resulted in a significant reduction in the number of patients that could have been included.

There was a small but not significant difference in the percentage of individuals with CRC in the *FLCN*^*MUT*^ and *FLCN*^*WT*^ group (3.3% vs 2.4%, *p* = 0.52) and the age at their first CRC (61.3 vs 69.8). The difference in age may be due to increased surveillance in patients with BHD, leading to an earlier diagnosis of CRC. The relatively smaller size of their CRCs might be a reflection of this (39.8 vs 46.7 mm). Interestingly, the youngest patient with CRC, who was in the *FLCN*^*MUT*^ group, underwent the colonoscopy because of skin lesions and a presumed increased risk for CRC. Another factor to consider when interpreting these results is that more colonoscopies in the *FLCN*^*MUT*^ group might have prevented the development of CRC. Adenomas are considered pre-cancerous lesions, as opposed to hyperplastic polyps.^29^ A small proportion of adenomas progresses to carcinoma over a time period of approximately 10-15 years, so colonoscopy with removal of visible polyps, reduces the risk of developing CRC.^30,31^ On the other hand, the number of high-risk polyps that were removed in the *FLCN*^*MUT*^ group was not higher than in the *FLCN*^*WT*^ group. Finding an adenoma with villous histology, high grade dysplasia and/or larger size increases the risk for a future CRC.^32^ The proportion of villous adenomas and adenomas with high grade dysplasia in the *FLCN*^*MUT*^ group was comparable to the *FLCN*^*WT*^ group. The size of the polyps was often not mentioned in the summary of the pathology report, so we could not evaluate this factor. Significantly more individuals in the *FLCN*^*MUT*^ group were diagnosed with at least 1 polyp (11.8% vs 5.8%, *p* = 0.004). If patients with BHD are more prone to develop colon polyps, we would have expected that individuals in the *FLCN*^*MUT*^ group had more polyps per person than the individuals in the *FLCN*^*WT*^ group. This was not the case, so we hypothesised that the difference between polyp incidences is likely to have been caused by a surveillance bias, since some patients with BHD are advised to undergo surveillance colonoscopies whereas their family members without BHD are not. Individuals without BHD will only undergo colonoscopy in case of situations unrelated to BHD such as a positive CRC population screening test or gastro-intestinal symptoms. To further analyse this bias, we assessed the indications for colonoscopies in the *FLCN*^*MUT*^ group. For only 18 BHD patients, the indication for (the first) colonoscopy and detection of all polyps was known: in 6/18 patients (33%), the colonoscopies were performed as surveillance for CRC per BHD surveillance guidelines. It is plausible to assume that the polyps in these six patients would not have been detected if they had not been diagnosed with BHD. When extrapolating this observation to the whole group of BHD patients in this study, 33% of the BHD patients with at least one polyp (*n* = 16) would have underwent their colonoscopies because of BHD surveillance guidelines, meaning that these 16 BHD patients would not have had polyps detected without their BHD diagnosis. Removing these 16 from the total of 47 BHD patients with polyps, leaves us with 31 patients with at least one polyp in the *FLCN*^*MUT*^ group (7.8%). Compared to 5.8% in the *FLCN*^*WT*^ group, this is no longer a significant difference anymore (*p* = 0.32). This is a rough estimate, but might be another indication that the significant difference in individuals with polyps is likely to be caused by a higher number of colonoscopies in patients with BHD. Another possible bias could have occurred if patients from BHD families with a history of colorectal polyps are more likely to opt for predictive DNA testing. However, in our experience colorectal polyps have never been mentioned by any of our patients to be the reason for predictive testing or referral.

We did not confirm the putative genotype-phenotype correlation for mutations in the poly (C)_8_ tract. The percentage of individuals with polyps in this subgroup was even slightly lower compared to the whole *FLCN*^*MUT*^ group. Unlike an earlier report, none of the 24 individuals in this subgroup had a history of CRC.^11^

In conclusion, our data do not provide evidence for an increased risk for CRC in BHD, in line with the observations of a previous cohort study. If there is an association, it is likely to be small and it is doubtful whether surveillance with colonoscopy is indicated. Although it would be preferable to verify our observations by a prospective cohort study, based on our findings and the existing data from previous cohorts, we suggest to no longer advise patients with BHD to undergo surveillance by colonoscopy, unless indicated by a family history of CRC, in accordance with local guidelines. On the tumour level, the role of *FLCN* in CRCs in patients with BHD requires further exploration.

## Supplementary information

Supplementary Information

## Data Availability

Extensive data on individuals are supplied in the supplementary tables. The dataset used in the current study can be made available in a data repository, if wished for.

## References

[1] Zbar B, Alvord WG, Glenn G, Turner M, Pavlovich CP, Schmidt L (2002). Risk of renal and colonic neoplasms and spontaneous pneumothorax in the Birt-Hogg-Dube syndrome. Cancer Epidemiol. Biomarkers Prev..

[2] Birt AR, Hogg GR, Dube WJ (1977). Hereditary multiple fibrofolliculomas with trichodiscomas and acrochordons. Arch. Dermatol..

[3] Toro JR, Glenn G, Duray P, Darling T, Weirich G, Zbar B (1999). Birt-Hogg-Dube syndrome: a novel marker of kidney neoplasia. Arch. Dermatol..

[4] Nickerson ML, Warren MB, Toro JR, Matrosova V, Glenn G, Turner ML (2002). Mutations in a novel gene lead to kidney tumors, lung wall defects, and benign tumors of the hair follicle in patients with the Birt-Hogg-Dube syndrome. Cancer Cell.

[5] Hornstein OP, Knickenberg Perifollicular M (1975). fibromatosis cutis with polyps of the colon-a cutaneo-intestinal syndrome sui generis. Arch. Dermatol. Res..

[6] Schachtschabel, A. A., Kuster, W. & Happle, R. [Perifollicular fibroma of the skin and colonic polyps: Hornstein-Knickenberg syndrome]. *Hautarzt* **47**, 304–306 (1996). 10.1007/s001050050420 8655317

[7] Khoo SK, Giraud S, Kahnoski K, Chen J, Motorna O, Nickolov R (2002). Clinical and genetic studies of Birt-Hogg-Dube syndrome. J. Med. Genet..

[8] Rongioletti F, Hazini R, Gianotti G, Rebora A (1989). Fibrofolliculomas, tricodiscomas and acrochordons (Birt-Hogg-Dube) associated with intestinal polyposis. Clin. Exp. Dermatol..

[9] Dodds T, Delprado W, Meagher A, Tucker K, Earls Colorectal P (2016). carcinoma with an oncocytic component occurring in a patient with Birt-Hogg-Dube syndrome. Pathology.

[10] Kluger N, Giraud S, Coupier I, Avril MF, Dereure O, Guillot B (2010). Birt-Hogg-Dube syndrome: clinical and genetic studies of 10 French families. Br. J. Dermatol..

[11] Nahorski MS, Lim DH, Martin L, Gille JJ, McKay K, Rehal PK (2010). Investigation of the Birt-Hogg-Dube tumour suppressor gene (FLCN) in familial and sporadic colorectal cancer. J. Med. Genet..

[12] Toro JR, Wei MH, Glenn GM, Weinreich M, Toure O, Vocke C (2008). BHD mutations, clinical and molecular genetic investigations of Birt-Hogg-Dube syndrome: a new series of 50 families and a review of published reports. J. Med. Genet..

[13] Schmidt LS, Nickerson ML, Warren MB, Glenn GM, Toro JR, Merino MJ (2005). Germline BHD-mutation spectrum and phenotype analysis of a large cohort of families with Birt-Hogg-Dube syndrome. Am. J. Hum. Genet..

[14] Ionov Y, Peinado MA, Malkhosyan S, Shibata D, Perucho M (1993). Ubiquitous somatic mutations in simple repeated sequences reveal a new mechanism for colonic carcinogenesis. Nature.

[15] Thibodeau SN, Bren G, Schaid D (1993). Microsatellite instability in cancer of the proximal colon. Science.

[16] Aaltonen LA, Peltomaki P, Leach FS, Sistonen P, Pylkkanen L, Mecklin JP (1993). Clues to the pathogenesis of familial colorectal cancer. Science.

[17] Shin JH, Shin YK, Ku JL, Jeong SY, Hong SH, Park SY (2003). Mutations of the Birt-Hogg-Dube (BHD) gene in sporadic colorectal carcinomas and colorectal carcinoma cell lines with microsatellite instability. J. Med. Genet..

[18] Kahnoski K, Khoo SK, Nassif NT, Chen J, Lobo GP, Segelov E (2003). Alterations of the Birt-Hogg-Dube gene (BHD) in sporadic colorectal tumours. J. Med. Genet..

[19] Vocke CD, Yang Y, Pavlovich CP, Schmidt LS, Nickerson ML, Torres-Cabala CA (2005). High frequency of somatic frameshift BHD gene mutations in Birt-Hogg-Dube-associated renal tumors. J. Natl Cancer Inst..

[20] Fujii H, Jiang W, Matsumoto T, Miyai K, Sashara K, Ohtsuji N (2006). Birt-Hogg-Dube gene mutations in human endometrial carcinomas with microsatellite instability. J. Pathol..

[21] Jiang, W., Fujii, H., Matsumoto, T., Ohtsuji, N., Tsurumaru, M. & Hino Birt-Hogg-Dube, O. (BHD) gene mutations in human gastric cancer with high frequency microsatellite instability. *Cancer Lett.* **248**, 103–111 (2007). 10.1016/j.canlet.2006.06.005 16870330

[22] da Silva NF, Gentle D, Hesson LB, Morton DG, Latif F, Maher ER (2003). Analysis of the Birt-Hogg-Dube (BHD) tumour suppressor gene in sporadic renal cell carcinoma and colorectal cancer. J. Med. Genet..

[23] Guda K, Veigl ML, Varadan V, Nosrati A, Ravi L, Lutterbaugh J (2015). Novel recurrently mutated genes in African American colon cancers. Proc. Natl Acad. Sci. USA.

[24] Boman P, Ousager L, Friis-Hansen L, van Overeem Hansen T, Broesby-Olsen S, Gerdes Is A (2014). colorectal neoplasia part of the Birt-Hogg-Dubé syndrome?. J. Gastroenterol. Hepatol. Res..

[25] Menko FH, van Steensel MA, Giraud S, Friis-Hansen L, Richard S, Ungari S (2009). Birt-Hogg-Dube syndrome: diagnosis and management. Lancet Oncol..

[26] Vereniging Klinische Genetica Nederland, W.K.O., Erfelijke en familiaire tumoren: richtlijnen voor diagnosiek en preventie. (Vereniging Klinische Genetica, Nederland, 2017)

[27] Casparie M, Tiebosch AT, Burger G, Blauwgeers H, van de Pol A, van Krieken JH (2007). Pathology databanking and biobanking in The Netherlands, a central role for PALGA, the nationwide histopathology and cytopathology data network and archive. Cell Oncol..

[28] Rex DK, Petrini JL, Baron TH, Chak A, Cohen J, Deal SE (2006). Quality indicators for colonoscopy. Am. J. Gastroenterol..

[29] Rex DK, Ahnen DJ, Baron JA, Batts KP, Burke CA, Burt RW (2012). Serrated lesions of the colorectum: review and recommendations from an expert panel. Am. J. Gastroenterol..

[30] Winawer SJ, Zauber AG, Ho MN, O'Brien MJ, Gottlieb LS, Sternberg SS (1993). Prevention of colorectal cancer by colonoscopic polypectomy. The National Polyp Study Workgroup. N. Engl. J. Med..

[31] Muto T, Bussey HJ, Morson BC (1975). The evolution of cancer of the colon and rectum. Cancer.

[32] Calderwood AH, Lasser KE, Roy HK (2016). Colon adenoma features and their impact on risk of future advanced adenomas and colorectal cancer. World J. Gastrointest. Oncol..

