# Supplementary material for: No evidence for increased prevalence of colorectal carcinoma in 399 Dutch patients with Birt-Hogg-Dubé syndrome
Source: Br J Cancer. 2019 Dec 20;122(4):590–4. doi: 10.1038/s41416-019-0693-1 (PMC7028712; doi:10.1038/s41416-019-0693-1)
Supplement: Supplementary file 1 — Supplementary Information [file 41416_2019_693_MOESM1_ESM.pdf]

## Supplementary Information

**Figure S1**: dot plots of age (A) and diameter (B) of the first CRC

**Figure S2**: dot plots of the number of polyps per person (A) and age at first polyp (B)

**Table S1**: CRC and polyps in the $FLCN^{MUT}$ group

**Table S2**: CRC and polyps in the $FLCN^{WT}$ group

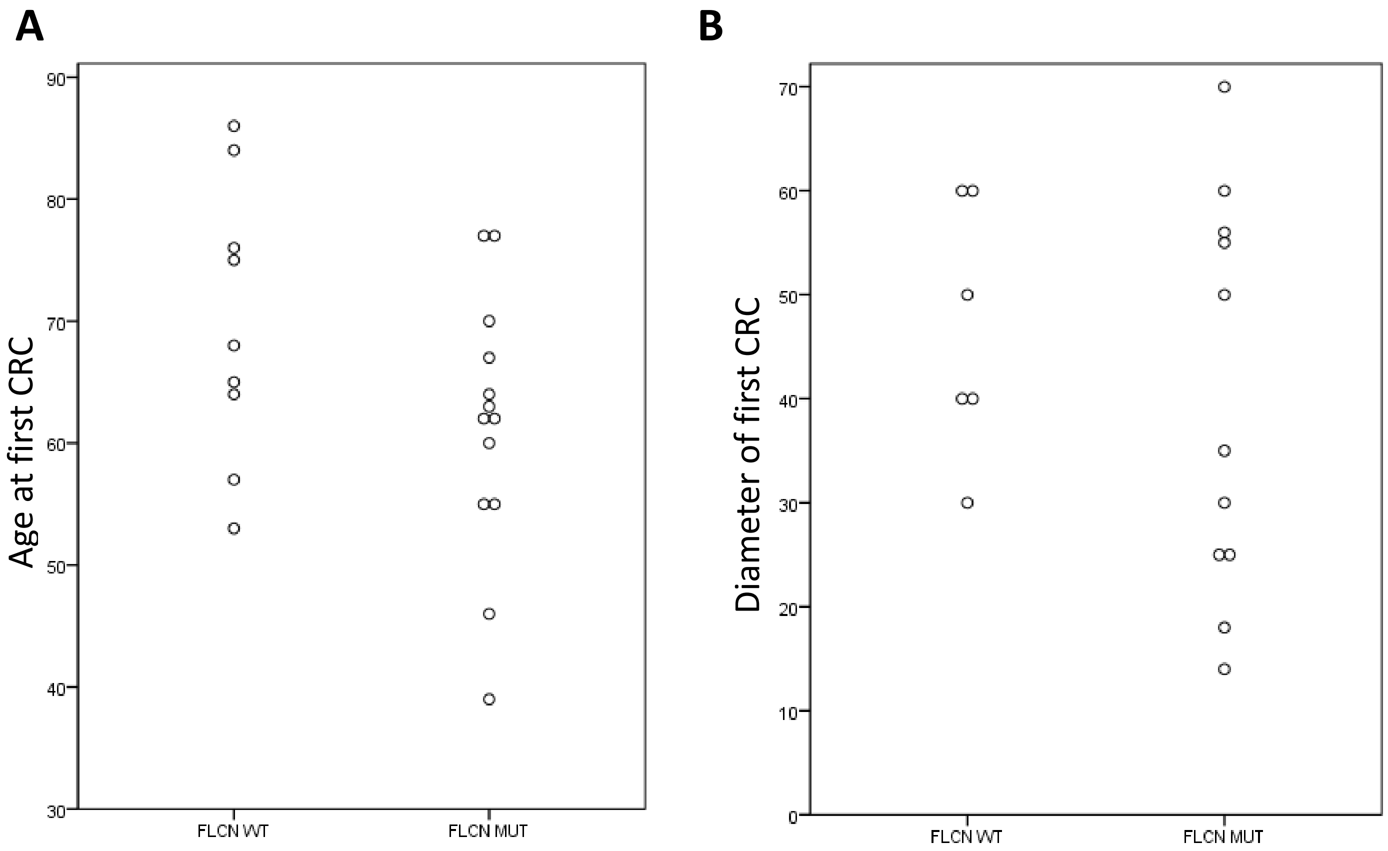


Figure S1: dot plots of age (A) and diameter (B) of the first CRC

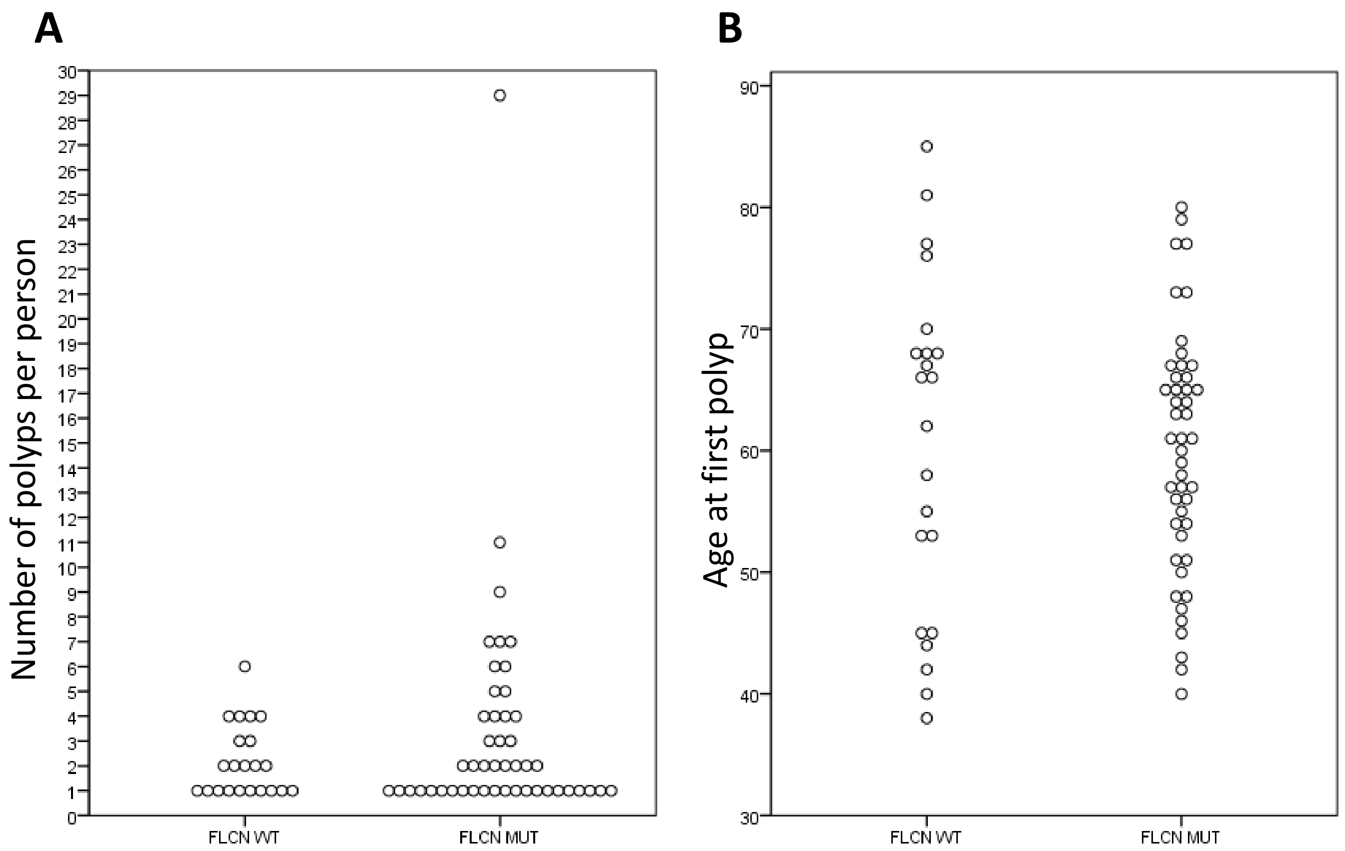


Figure S2: dot plots of the number of polyps per person (A) and age at first polyp (B)

| Table S1: CRC and polyps in the $FLCN^{MUT}$ group | | | | | | | | | | | | | | | | | | | | |
|---|---|---|---|---|---|---|---|---|---|---|---|---|---|---|---|---|---|---|---|---|
| **General** | | **CRC** | | | | | **Polyps** | | | | | | | | | | | | | |
| **Number** | **poly $(C)_8$** | **CRC (n)** | **Age** | **Location** | **Differentiation** | **Diameter** | **Polyp (n)** | **Age first** | **Location (n)** | | | **Grade of dysplasia (n)** | | | | **Histology (n)** | | | | |
| | | | | | | | | | *D* | *P* | *U* | *N/M* | *M* | *S* | *U* | *A* | *SS* | *H* | *U* |
| **1** | N | Y (1) | 62 | D | M | 18 | N | | | | | | | | | | | | |
| **2** | N | N | | | | | Y (1) | 65 | 1 | | | 1 | | | | 1 | | | |
| **3** | N | Y (1) | 46 | | M | 70 | Y (7) | 50 | 1 | 4 | 2 | 4 | | 1 | 2 | 5 | | 2 | |
| **4** | N | N | | | | | Y (3) | 43 | 1 | | 2 | 2 | | | 1 | 2 | | 1 | |
| **5** | N | N | | | | | Y (2) | 80 | 1 | | 1 | | | | 2 | 1 | | 1 | |
| **6** | N | N | | | | | Y (1) | 65 | 1 | | | 1 | | | | 1 | | | |
| **7** | N | Y (1) | 64 | P | M | 60 | Y (2) | 60 | 1 | | 1 | | | | 2 | | | 2 | |
| **8** | N | N | | | | | Y (1) | 53 | | 1 | | 1 | | | | 1 | | | |
| **9** | N | N | | | | | Y (3) | 57 | 1 | 2 | | 3 | | | | 2 | | 1 | |
| **10** | N | N | | | | | Y (5) | 67 | 4 | 1 | | 3 | | 1 | 1 | 4 | | 1 | |
| **11** | N | N | | | | | Y (1) | 48 | 1 | | | | | | 1 | | | 1 | |
| **12** | N | N | | | | | Y (6) | 64 | 2 | 4 | | 3 | | | 3 | 3 | | 3 | |
| **13** | N | Y (1) | 77 | P | W/M | 30 | Y (1) | 77 | | | 1 | 1 | | | | 1 | | | |
| **14** | N | Y (1) | 39 | D | M | 35 | N | | | | | | | | | | | | |
| **15** | Y | N | | | | | Y (1) | 42 | | | 1 | | 1 | | | 1 | | | |
| **16** | N | Y (1) | 55 | D | M | 56 | Y (1) | 66 | | | 1 | 1 | | | | | | 1 | |
| **17** | N | N | | | | | Y (2) | 57 | 2 | | | | | | 2 | | | 2 | |
| **18** | N | Y (1) | 62 | D | M | 50 | Y (11) | 57 | 2 | 3 | 6 | 3 | | | 8 | 3 | | 8 | |
| **19** | N | N | | | | | Y (1) | 56 | | | 1 | 1 | | | | 1 | | | |
| **20** | N | N | | | | | Y (6) | 46 | 1 | 1 | 4 | 1 | 2 | | 3 | 3 | | 3 | |
| **21** | N | N | | | | | Y (9) | 64 | 5 | 4 | | 7 | | | 2 | 6 | 1 | 2 | |
| **22** | N | N | | | | | Y (1) | 68 | 1 | | | | | 1 | | 1 | | | |
| **23** | N | N | | | | | Y (1) | 65 | | | 1 | 1 | | | | | | 1 | |
| **24** | N | N | | | | | Y (2) | 79 | | 1 | 1 | 2 | | | | 2 | | | |
| **25** | N | N | | | | | Y (4) | 61 | 2 | 1 | 1 | 1 | 1 | | 2 | 2 | | 2 | |
| **26** | N | N | | | | | Y (1) | 54 | | 1 | | 1 | | | | 1 | | | |
| **27** | N | Y (1) | 60 | D | M | 14 | N | | | | | | | | | | | | |
| **28** | N | N | | | | | Y (1) | 58 | 1 | | | | | | 1 | | | 1 | |
| **29** | N | N | | | | | Y (1) | 65 | | 1 | | 1 | | | | 1 | | | |
| **30** | N | N | | | | | Y (2) | 51 | | 1 | 1 | 1 | | 1 | | 2 | | | |

| | | | | | | | | | | | | | | | | | | | |
|---|---|---|---|---|---|---|---|---|---|---|---|---|---|---|---|---|---|---|---|
| **31** | N | N | | | | | Y (5) | 73 | 1 | 3 | 1 | 5 | | | | 5 | | | |
| **32** | N | Y (1) | 77 | | W/M | 55 | Y (4) | 77 | 1 | | 3 | 3 | | | 1 | 3 | | 1 | |
| **33** | N | N | | | | | Y (7) | 61 | | | 7 | 6 | | | 1 | 5 | 1 | 1 | |
| **34** | Y | N | | | | | Y (2) | 47 | | 2 | | 2 | | | | 2 | | | |
| **35** | N | N | | | | | Y (2) | 69 | 2 | | | 2 | | | | 2 | | | |
| **36** | N | N | | | | | Y (7) | 54 | 4 | 2 | 1 | 2 | | 1 | 4 | 3 | | 4 | |
| **37** | N | N | | | | | Y (1) | 66 | | 1 | | 1 | | | | 1 | | | |
| **38** | N | N | | | | | Y (1) | 61 | | | 1 | 1 | | | | | | | 1 |
| **39** | N | N | | | | | Y (3) | 67 | | | 3 | 2 | 1 | | | 3 | | | |
| **40** | N | N | | | | | Y (1) | 45 | | 1 | | 1 | | | | 1 | | | |
| **41** | N | Y (1) | 55 | D | M | | Y (1) | 63 | 1 | | | 1 | | | | 1 | | | |
| **42** | N | N | | | | | Y (1) | 51 | 1 | | | 1 | | | | 1 | | | |
| **43** | N | N | | | | | Y (2) | 48 | 1 | 1 | | 1 | | | 1 | 1 | | 1 | |
| **44** | N | N | | | | | Y (1) | 40 | 1 | | | | 1 | | | 1 | | | |
| **45** | N | Y (1) | 67 | D | | | Y (4) | 67 | 2 | 1 | 1 | 3 | | | 1 | 3 | | 1 | |
| **46** | N | N | | | | | Y (4) | 55 | 1 | 1 | 2 | | | | 4 | 3 | | 1 | |
| **47** | N | N | | | | | Y (1) | 73 | 1 | | | | | | 1 | | | 1 | |
| **48** | N | N | | | | | Y (1) | 56 | | 1 | | | | | 1 | | 1 | | |
| **49** | N | Y (2) | 63 (both) | P (both) | W/M + U | 25 & 10 | Y (29) | 63 | | 1 | 28 | 13 | | | 16 | 13 | 1 | 15 | |
| **50** | N | Y (1) | 70 | D | W/M | 25 | N | | | | | | | | | | | | |
| **51** | N | N | | | | | Y (1) | 59 | | | 1 | 1 | | | | 1 | | | |

N, no; Y, yes; U, unknown. Location: D, distal; P, proximal. Differentiation: M, moderate; W/M, well/moderate; P, poor. Grade of dysplasia: N/M, no/mild; M, moderate; S, severe. Histology: A, adenoma; SS, sessile serrated adenoma; H, hyperplastic polyp.

**Table S2: CRC and polyps in the FLCN$^{WT}$ group**

| General | CRC | | | | | Polyps | | | | | | | | | | | | |
|---|---|---|---|---|---|---|---|---|---|---|---|---|---|---|---|---|---|---|
| **Number** | **CRC (n)** | **Age** | **Location** | **Differentiation** | **Diameter** | **Polyp (n)** | **Age first** | **Location (n)** | | | **Grade of dysplasia (n)** | | | | **Histology (n)** | | | |
| | | | | | | | | *D* | *P* | *U* | *N/M* | *M* | *S* | *U* | *A* | *SS* | *H* | *U* |
| **52** | N | | | | | Y(1) | 45 | *1* | | | | | | *1* | | | *1* | |
| **53** | N | | | | | Y(1) | 81 | 1 | | | | | 1 | | 1 | | | |
| **54** | Y(1) | 76 | D | W/M | 40 | Y(4) | 76 | 2 | 1 | 1 | 2 | | | 2 | 1 | 1 | 2 | |
| **55** | N | | | | | Y(4) | 66 | 2 | 2 | | 3 | | | 1 | 1 | 2 | 1 | |
| **56** | N | | | | | Y(2) | 38 | 1 | 1 | | 2 | | | | 2 | | | |
| **57** | Y(1) | 64 | D | M | | N | | | | | | | | | | | | |
| **58** | N | | | | | Y(2) | 40 | 1 | 1 | | 2 | | | | 1 | | 1 | |
| **59** | N | | | | | Y(1) | 68 | | | 1 | | 1 | | | 1 | | | |
| **60** | Y(1) | 84 | D | P | 60 | Y(1) | 85 | | 1 | | | | | 1 | 1 | | | |
| **61** | N | | | | | Y(2) | 53 | 1 | | 1 | 1 | | | 1 | 1 | | 1 | |
| **62** | N | | | | | Y(1) | 67 | 1 | | | 1 | | | | 1 | | | |
| **63** | Y(2) | 53 & 65 | D | M (both) | U & 30 | Y(3) | 58 | | 2 | 1 | 1 | 2 | | | 3 | | | |
| **64** | N | | | | | Y(1) | 66 | | 1 | | 1 | | | | 1 | | | |
| **65** | N | | | | | Y(1) | 68 | | 1 | | 1 | | | | 1 | | | |
| **66** | Y(1) | 65 | P | W/M | U | N | | | | | | | | | | | | |
| **67** | N | | | | | Y(1) | 62 | | | 1 | 1 | | | | 1 | | | |
| **68** | N | | | | | Y(4) | 45 | 2 | 1 | 1 | 3 | 1 | | | 4 | | | |
| **69** | Y(1) | 57 | P | M | 40 | N | | | | | | | | | | | | |
| **70** | Y(1) | 86 | P | M | 50 | Y(6) | 70 | | 1 | 5 | 5 | | | 1 | 5 | | 1 | |
| **71** | N | | | | | Y(1) | 55 | 1 | | | | | | 1 | | | 1 | |
| **72** | N | | | | | Y(4) | 77 | 2 | | 2 | 1 | | 1 | 2 | 2 | | 2 | |
| **73** | Y(1) | 75 | P | | 30 | N | | | | | | | | | | | | |
| **74** | N | | | | | Y(2) | 42 | | | 2 | 1 | | | 1 | 1 | | 1 | |
| **75** | Y(1) | 68 | D | M | 60 | Y(2) | 68 | 1 | | 1 | 1 | | | 1 | 1 | | 1 | |
| **76** | N | | | | | Y(1) | 44 | 1 | | | | | | 1 | | | 1 | |
| **77** | N | | | | | Y(3) | 53 | | | 3 | 2 | | | 1 | 2 | | 1 | |

N, no; Y, yes; U, unknown. Location: D, distal; P, proximal. Differentiation: M, moderate; W/M, well/moderate; P, poor. Grade of dysplasia: N/M, no/mild; M, moderate; S, severe. Histology: A, adenoma; SS, sessile serrated adenoma; H, hyperplastic polyp.
